# Current Developments in the Management of Amblyopia with the Use of Perceptual Learning Techniques

**DOI:** 10.3390/medicina60010048

**Published:** 2023-12-27

**Authors:** Konstantinos T Tsaousis, Georgios Mousteris, Vasilios Diakonis, Stergios Chaloulis

**Affiliations:** 1 General Hospital of Volos, 382 22 Volos, Greece; 2 2nd Ophthalmology Department, Metropolitan Hospital, 185 47 Athens, Greece

**Keywords:** neuroplasticity, interocular suppression, visual perceptual learning, dichoptic therapy, binocular vision treatment, virtual reality

## Abstract

*Background and Objectives*: Amblyopia is a neurodevelopmental disorder caused by interocular suppression of visual input, affecting visual acuity, stereopsis, contrast sensitivity, and other visual functions. Conventional treatment comprises occlusion of the sound eye. In recent years, novel therapies that deploy perceptual learning (PL) principles have been introduced. The purpose of this study is to assess the latest scientific data on this topic. *Materials and Methods*: For this purpose, we conducted a literature search for relevant studies published during the previous 4 years (2020–2023). *Results*: A plethora of visual perceptual learning protocols have been recently developed. Dichoptic video games, contrast rebalanced movies, and online perceptual training platforms are the main formats. Perceptual learning activates neuroplasticity, overcomes interocular suppression, and improves the visual impairments induced by amblyopia. *Conclusions:* This novel treatment is effective in both children and adults, as well as in patients non-responding to patching.

## 1. Introduction

### 1.1. Background

Amblyopia or “lazy eye” is defined as the reduction of the best corrected visual acuity (BCVA) of one or both eyes without the presence of any organic cause [1,2]. It is a neurodevelopmental disorder regarding abnormal cortical processing of visual input from both eyes that occurs during the critical period of visual system development, causing several visual defects [1,2,3]. It is the most common vision impairment in children, affecting up to 3.9% of the world population [1,2,4]. The main amblyogenic factors are: (a) Refractive errors, presented as anisometropia, which refers to a significant difference in refractive error between the two eyes, or as high ametropia, which refers to a high refractive error existing in both eyes; (b) strabismus, which is the misalignment of both eyes; and (c) deprivation of visual input, usually due to congenital cataract, eyelid ptosis, corneal opacities, limbal dermoids, or other ocular conditions preventing light from reaching the normal retina [2,5,6]. According to these causes, amblyopia is classified as refractive, strabismic, or deprivation amblyopia. Also, in some cases, amblyopia is due to a combined mechanism. Amblyopia is usually unilateral, and it is defined as visual acuity (VA) worse than 20/30 in an otherwise healthy eye, alongside an interocular visual acuity difference of at least two VA chart lines [1,7]. In bilateral amblyopia, both eyes have a reduced BCVA.

In children younger than 3 years old, strabismus is the most frequent cause of amblyopia (above 80% of cases), followed by mixed mechanism (13%) and refractive error (5%) [8]. In children <7 years old, strabismus and anisometropia are almost equally responsible for amblyopia (38% and 37%, respectively), while the rest of amblyopia cases are due to combined mechanisms [9]. According to other authors, deprivation of visual stimuli accounts for 4% of amblyopia cases [1].

The definition of amblyopia describes just the tip of the iceberg, as it affects a plethora of monocular and binocular functions besides VA. In further detail, amblyopia causes vulnerability to crowding (reduced ability to identify and distinguish a certain object within a pile of objects), poor stereopsis (depth perception), impaired contrast sensitivity (CS), spatial localization, form and motion perception, fixation instability, selective visual attention deficits as well as visuomotor coordination deficits, with great impact on everyday activities [1,2,3].

Perception of depth is a complex process where the brain integrates many different visual cues, some of which are derived from each eye (non-binocular cues), while other information is derived by the summation of visual input from both eyes (binocular cues) [3]. Non-binocular cues include overlapping of objects, perspective-conical projection of objects within distance, and relative size of objects within a scene, as well as lighting and shading over objects [3]. All these cues are essential sensory inputs for developing visuomotor skills during childhood [3,10]. Therefore, discordant binocular experience in early childhood, as is the case with amblyopic individuals, could affect the optimal development of eye-hand coordination. Accurate localization of objects within three-dimensional (3D) space is essential for developing the ability to plan and execute the required hand movements to reach and grasp a target object effectively [3,10]. Neuroimaging studies have shown reduced responses in the cortical regions responsible for depth perception in amblyopic patients [11].

Not all etiological factors have equal amblyogenic potential. For example, astigmatism is known to induce deeper amblyopia compared to hyperopia of equal diopters. Furthermore, strabismus is reported to have a stronger impact on visual functions than anisometropia [2,8,12]. Therefore, strabismic amblyopia patients present greater crowding effect, more severe loss of stereoacuity, greater contrast sensitivity deficits, especially in higher spatial frequencies and poorer performance eye-hand coordination in tasks [10,12]. Also, studies highlight that strabismus patients with amblyopia are less responsive to treatment and show higher regression rates after treatment cessation compared to anisometropia patients [12].

### 1.2. Pathogenesis

Amblyopia was previously perceived as a monocular disorder. Recent observations have changed this notion. It is now accepted that amblyopia is a result of binocular dysfunction [2,13,14].

Binocular perception is achieved in normally-sighted individuals by the integration of the input from both eyes in the visual cortex [11,14]. These incoming signals are similar enough to get correlated and fused together, and each eye contributes equally to the perceived image [11,14]. Binocularity is established promptly at birth through complex excitatory and inhibitive neuronal circuitry in the early visual cortex [11,15]. During early infancy, between 3 to 5 months of age, neural connections increase massively in this area [11,15]. During early childhood, these synapses constantly change, leading to either the strengthening of correct connections or the inhibition of incorrect connections [15]. Frequent use of a certain neural connection promotes synaptic strengthening, and the opposite happens to idle connections [15]. This process is defined as neuroplasticity. The time period where these important changes take place is referred to as the critical period, and for the development of vision, it is known to extend until 7 years of age [15]. Neuroplasticity beyond that period is thought to gradually decline until adulthood. Thus, children are most susceptible to developing amblyopia during the first 2–3 years of life, and the risk gradually decreases until the end of the critical period, when the visual system matures and retinocortical pathways become strongly established [15]. As recent studies revealed, the adult cortex still retains a considerable amount of neuroplasticity [15,16]. In amblyopic individuals, under binocular viewing conditions, the brain receives dissimilar and conflicting visual input from corresponding retinal points, which can cause confusion and diplopia [14]. In order to prevent this situation, the brain inhibits the information from the eye that is either receiving a blurry image (in anisometropia), becomes misaligned (in strabismus), or receives no input (in deprivation amblyopia) [14]. This mechanism is referred to as suppression in favor of the dominant eye, causing the “weaker” eye to become amblyopic [14]. The depth of suppression is positively associated with the amount of VA reduction [14,17]. Researchers, using specially designed dichoptic VA charts, showed that the amblyopic eye scored higher acuity when the fellow eye was occluded, but in binocular viewing conditions, it performed worse as a result of suppression from the dominant eye [17]. This effect was present in naive amblyopic and supposedly treated patients as well [17]. These findings support previous observations suggesting that amblyopia is indeed the result of binocular dysfunction.

Suppression is marked across the whole amblyopic visual field, but it is stronger in the foveal region, creating a distinct functionally blind area called suppression scotoma [14,18,19]. Luminance and contrast sensitivity thresholds are elevated within that area, form and motion information are suppressed, and objects presented within the scotoma do not become consciously aware [14,18,19]. Recent studies show that the information from the amblyopic eye, though strongly suppressed from conscious perception, still remains available for binocular processing [18].

Discordant visual signals from the two eyes have a major effect on the primary visual cortex V1 neurons during the early critical period of development, altering neural circuitry [11,14,16,20,21]. Beyond infancy, the effect of dissimilar visual input results in fixation instability, with the development of abnormal fixation eye movements (FEMs), eccentric fixation, and greater VA loss [2,20,22]. The presence of nystagmus in amblyopic patients is associated with more severe visual impairment [20].

As recent research data unveiled the role of binocular dysfunction in the etiology of amblyopia, treatment approaches have been shifted toward that direction [2,14,19]. Novel amblyopia therapies are based on the principles of perceptual learning (PL). PL can be defined as improved sensory perception or execution performance as a result of practice, achieved by repetitive exposure to certain stimuli or repetitive execution of certain physical tasks [16,23,24]. The basic principle behind the training effects of perceptual learning is that repetitive stimulation causes strong and synchronous activation of certain cellular populations along the neuronal pathway, mediating a specific neurosensory task and promoting synaptic strengthening between those neurons (Hebbian plasticity) [16]. The result is permanent neuroplastic changes through training, with consequent improvement in neurosensory response [14,16]. These principles applied in visual functions could be an effective treatment for amblyopia deficits. The effect of PL in shaping neural plasticity on the visual cortex and subsequent in rebalancing interocular dominance has been demonstrated in amblyopia patients through changes in the amplitude of steady-state visually evoked potentials (SSVEP) measured before and after a number of treatment sessions [25,26], while other researchers in an ongoing study aim to confirm the same effect by detecting changes in functional Magnetic Resonance Imaging (f-MRI) of treated patients [27].

## 2. Materials and Methods

### 2.1. Objective

The present literature review attempted to identify and evaluate studies pertaining to the latest advances in the treatment of amblyopia with perceptual learning techniques.

### 2.2. Eligibility Criteria

- Inclusion criteria:
  - Studies published in English;
  - Only studies performed in humans;
  - All types of amblyopia.
- Exclusion criteria:
  - Studies previous to the year 2020;
  - Articles not referring to perceptual learning as a treatment for amblyopia;
  - Review articles.

**Figure 1:**
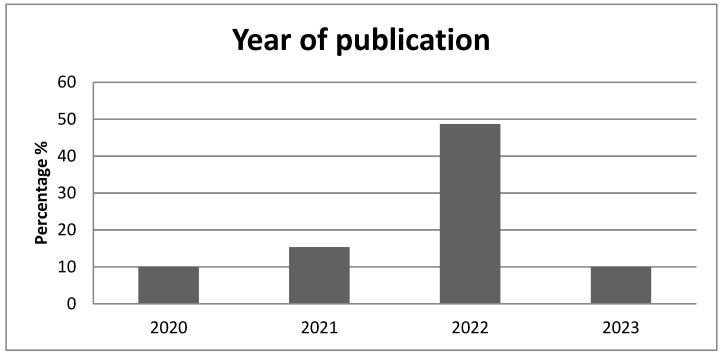
Year of publication of the studies included.

**Figure 2:**
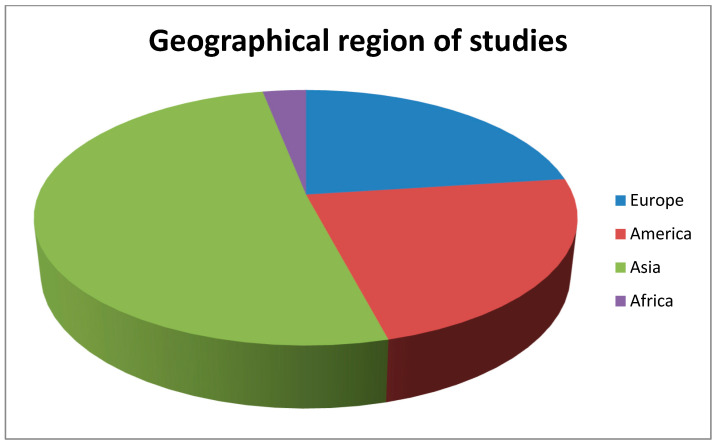
The geographical distribution of the studies included.

A literature search was performed in September 2023 in PubMed database using the terms “amblyopia, perceptual learning treatment” and “amblyopia, dichoptic treatment”. The search yielded 141 and 195 articles, respectively. The results were narrowed down to 104 and 149, respectively, by filtering out all non-human studies and those not written in English. We also excluded articles published prior to the year 2020, leaving a total of 87 (33 and 54, respectively) eligible studies. 17 duplicates were also removed. After initial screening and manual evaluation of abstracts, another 15 were removed as irrelevant to the topic. The remaining 39 articles were included in the present review. Besides clinical studies, we have included articles highlighting mechanisms behind the therapeutic effect of visual perceptual learning in the treatment of amblyopia. Although we have searched for all types of amblyopia, the vast majority of studies found are related to refractive and strabismic amblyopia and combined refractive/strabismic. There are only a few studies on perceptual learning treatment for deprivation amblyopia with rather small samples, which may limit the significance of their findings. Also, most of the studies found refer to the treatment of unilateral amblyopia, and only a small portion investigates bilateral amblyopia cases. Table 1 summarizes the clinical studies found in our literature search. Figure 1 shows the percentage of studies published per year, and Figure 2 shows the geographical distribution of the review’s studies. 

## 3. Results

### 3.1. Binocular Approaches

Traditional amblyopia treatment consists of occlusion (with opaque eye patches or Bangerter films) or penalization with atropine eye drops of the sound eye to train the amblyopic eye [61]. These methods have proven to be effective in improving VA and have been the gold standard for decades [62]. Amblyopia, though, as mentioned above, affects multiple visual functions, and occlusion does not restore these deficits [30].

Recent scientific data suggest that the conventional approach may be in the wrong direction. From a physiological aspect, it is fundamentally wrong to force a binocular visual system into monocular viewing in order to improve its function [2,13]. Furthermore, evidence shows that VA loss in amblyopic patients is probably the secondary consequence rather than the initial problem, which eventually is interocular imbalance and suppression of visual information from the amblyopic eye at the visual cortex [13,14,19]. While patching has proven to be effective in restoring VA, it does not alleviate other binocular deficits. Studies show that patients thought to be treated effectively, achieving normal VA, still exhibit significant impairment in contrast sensitivity and stereoacuity [62]. As already mentioned, the visual information from the amblyopic eye, though being suppressed, is still available for visual processing, contributing to binocular vision [18]. These observations suggest that amblyopia treatment should address the binocular dysfunction [13].

As mentioned above, novel amblyopia therapies are based on perceptual learning principles, and they are targeted toward binocular dysfunction and interocular suppression.

Various visual tasks like contrast detection, letter identification in noise, position discrimination in noise, Vernier acuity, and Gabor detection could serve as stimuli for PL amblyopia treatment. The digital progress allowed the development of a vast array of PL therapeutic tools that we will discuss in detail. PL could be executed in either a monocular or binocular fashion. PL treatments that we describe below are applied in patients consequently after initial refractive adaptation with spectacles for refractive amblyopia. As for strabismic and deprivation cases, PL is applied after surgical correction of the underlying cause.

#### 3.1.1. Monocular Perceptual Learning

This type of training is performed with the dominant eye occluded, and processed images are presented to the amblyopic eye in higher contrast to revert suppression [23,37,38]. A retrospective study investigated the effectiveness of monocular PL with the use of Amblyopia iNET training software (Amblyopia iNET, Home Therapy Systems Inc., Gold Canyon, AZ, USA) combined with patching, in comparison to patching alone [61]. Results showed an obvious impact of PL in VA and CS [37]. Another study reported similar improvement in VA and CS in patients treated with monocular PL over the critical period [38].

#### 3.1.2. Binocular Perceptual Learning

Binocular training programs involve the use of both eyes together. The most commonly used method is referred to as dichoptic therapy, where each eye is exposed to similar but slightly altered stimuli presented simultaneously in order to overcome interocular suppression and promote binocular summation [13,20,23,24,33]. Three different strategies have been utilized to achieve dichoptic presentation:

- Contrast rebalancing: The same image is presented in full contrast to the amblyopic eye and with significantly reduced contrast to the fellow eye [24,40];
- Visual blur: Presentation of identical images but with refractive blurring over the non-amblyopic eye [24,29,36];
- Partial presentation: Different elements of a visual scene are presented in each eye (as complementary parts of a puzzle), and the whole image can be perceived through binocular fusion [24].

Researchers over the years have designed various training programs to apply PL for visual rehabilitation in amblyopic patients. Most of them require the use of electronic equipment, and dichoptic viewing conditions are achieved with the use of polarizing or red/cyan color filters, selectively blocking information from each eye [28,41]. Furthermore, as a general rule, all training programs begin with details fully highlighted in favor of the amblyopic eye and fully faded for the sound eye, depending on the depth of amblyopia and, consequently, the interocular VA difference [12,13]. During the course of treatment, as patients overcome suppression and improve their performance, contrast is gradually rebalanced between the two eyes until equal values [12,13]. Below, we discuss the main types of visual PL therapies:

- Interactive video games;
- Virtual reality/augmented reality;
- Passive dichoptic movie/video viewing;
- Vision therapy;
*Transcranial electric stimulation (t-ES).

*Though t-ES is not a type of PL treatment, we mention it in this section because electric brain stimulation has been applied in combination with visual PL in several studies in order to enhance the training effects of PL by boosting neural excitability and neuroplasticity [63].

##### Interactive Video Games

One very popular form of PL is interactive video games, where different elements of the game are presented in each eye, and the player has to combine information from both eyes to interact accordingly and fulfill the game task. These games are designed as software applications either installed on a computer, tablet, smartphone, or game console or played online [25,27,31,33,39,42,43,44,63]. Several studies evaluated the effectiveness of dichoptic video games for the treatment of amblyopia. Researchers have developed a variety of original dichoptic video games or modified pre-existing commercial ones that could be played on various devices or training platforms, as we explain further.

- Occlu-tab (or Occlu-pad): Occlutab (Yaguchi Electric Co., Ltd., Ishinomaki, Japan) (or Occlupad as it was alternatively named in a few countries) is a modified i-Pad tablet device with the addition of a linear polarization filter on the display resulting in a “white screen” appearance to the naked eye. Patients use the device to play various video games while wearing specially designed polarizing glasses, which make the game elements visible only to the amblyopic eye while the fellow eye can see a white screen. The glasses have circular polarization filters so that the amblyopic eye can still see the image displayed, even if patients rotate the tablet or tilt their heads [33]. The game tasks require the use of a palm-sized tangible block (like a touch mouse), promoting simultaneous training of eye-hand coordination skills [33]. Two randomized control studies (RCTs), with more than 200 amblyopic children in total, have compared the effectiveness of this videogame treatment in combination with refractive correction versus either corrective spectacles alone or eye patching, respectively [33,42]. Both yielded a significantly greater improvement in VA in the game group [33,42]. One study included children 4 to 6 years old, while the other included patients within and beyond the critical period (up to 12 years old).
- i-Pod: Another RCT investigated the treatment effect of a modified “Tetris” falling blocks game loaded on an i-Pod touch device in older patients (>7 years old, including adults) [39]. Selective-dichoptic presentation of the game elements was achieved by depicting the falling blocks with different colors from the bottom-lying blocks and with the use of red-green filter glasses in the study group. The training sessions began with the presentation of stimuli in maximum contrast to the amblyopic eye and reduced contrast to the sound eye in order to overcome interocular suppression. During the training course, the contrast for the fellow eye gradually increased. Results showed a significant improvement in VA in the study group, with additional improvement in stereoacuity [39]. Similar results have been reported in a case–control study with adult participants playing a dichoptic Tetris game on a stock i-Pad tablet device [34].

In contrast to these promising results, an older non-inferiority study with approximately 400 participants compared the effectiveness of the “Tetris i-Pad treatment to traditional patching [64]. The authors reported an inferior VA improvement in the binocular treatment group [64].

- Online PL platforms: Other treatment protocols consist of network-based training platforms, where patients perform online PL tasks from their home computer, and the training data and progression rates are stored online and could be remotely monitored by the clinician [28,45]. These platforms include appropriate algorithms to generate tailor-made training plans according to each patient’s age, refraction, baseline VA and stereoacuity, and adjust the plan according to their progress [28,65]. The patients wear filter glasses during the sessions while the software is presenting 3D images or random-dot anaglyphic stimuli intended to stimulate stereoscopic perception [28,45]. These perceptual games were proven effective in children aging within and beyond the critical period, resulting in VA and stereoacuity improvement, with stable results throughout a 6 month follow-up [28,45].

Other research groups designed PL games that target other visual functions impaired in amblyopic patients, like contrast discrimination and selective visual attention [25,31,46]. The game tasks required the patient to provide certain visual attention in order to search and count targets among distractors presented to the amblyopic and the fellow eye, respectively [25,31]. This therapeutic approach was tested in adults and in older children not responding to conventional treatment [25,31,43,46]. These studies concluded that dichoptic video games, besides improving VA, had additional benefits in stereoacuity, selective visual attention, and maximal tolerable visual noise contrast, depending on their training task [25,31,46]. In one of these studies, though authors describe the improvement of visual functions in patients unresponsive to previous patching as insignificant, they emphasize that these patients did gain better binocular summation and reading speed [43].

Finally, a small sample randomized control study with deprivation amblyopia participants versus healthy subjects evaluated the effectiveness of an online PL training program with adjustable low-contrast Gabor stimuli hidden between high-contrast Gabors, performed in both office and home sessions [5]. One branch of the amblyopic patients underwent a combined treatment of PL and subsequent patching in each session. Results showed an improvement in both VA and CS after a long-term treatment period (6 months) [5].

##### Virtual Reality/Augmented Reality

Virtual reality (VR) is the presentation of a simulated 3D environment generated by a computer, projected before the patient’s eyes on a head-mounted display [47]. It offers the patient a vivid, fully immersive experience, where he can actively interact with the software through multiple sensory functions, visual, auditory, and haptic [12,27,35,47,48]. Augmented reality (AR), on the other hand, is a live presentation of the real-world environment enhanced with additional digital elements projected over it [47].

Over recent years, VR and AR headset devices have become more commercially available, allowing the use of this technology in PL therapies with dichoptic video games for amblyopia [12,48]. VR/AR helmets provide ideal dichoptic viewing conditions while they offer the ability to adjust via software, proper alignment of the presented images in cases of strabismus, as well as other treatment variables, like contrast balance between the eyes and depth cues given within the games [12]. Both types (VR and AR) techniques foster concentration on the content displayed, as the helmets provide a wide field view at a close distance from the eyes, isolating completely all external stimuli [12].

A large study investigated the therapeutic effect of both VR and AR on a sample of 145 amblyopic children [35]. Researchers applied short-duration sessions (20 min) of PL games in order to excite cortical plasticity. Results showed a significant improvement in VA, stereoacuity and CS in both VR and AR groups [35]. An interesting finding was that CS in the AR group improved in all spatial frequencies, while it improved only in certain frequencies in the VR group [35]. Thus, AR seems to be more beneficial compared to VR in restoring contrast discrimination [35]. These results are consistent with another study that evaluated VR game visual therapy in adults with both normal and impaired vision [12]. Researchers developed two VR video games incorporating monocular and binocular depth cues in order to train and enhance stereoacuity. Both groups of participants showed in-game improvement in stereoscopic performance [12]. Similar results were also presented in a prospective pilot study that enrolled 90 patients between 4–12 years old with all types of amblyopia [49]. After 3 months course of VR treatment with a training software from Luminopia (Luminopia Inc., Cambridge, MA, USA), patients exhibited a significant improvement in VA (1.5 LogMAR lines) and stereoacuity [49]. Another study that compared the treatment effect of VR video games with occlusion showed greater VA improvement in children between 4–12 years old, suggesting a 15-fold higher efficiency in favor of VR treatment [50].

Additionally, a Hungarian study based on an AR platform with both pediatric and adult participants also yielded successful outcomes. Researchers designed a game called Stereopia, that was built on Leonar3Do equipment (Leonar3Do International Inc., Budapest, Hungary) [51]. In this setting, patients do not wear AR goggles but polarizing glasses, and the game is presented on a passive 3D display. Each eye viewed a stereo counterpart of the same game element on a dark background. A 3D handheld mouse enabled real-time manipulation of objects [49]. At the end of the training course, both age groups had significant benefits in stereoacuity, near visual acuity, and CS [51].

Finally, an ongoing RCT with 60 naive pediatric participants aims to compare the effectiveness of a VR platform named NEIVATECH against traditional patching. Researchers intend to verify the impact of the treatment on neuronal plasticity in the visual cortex by performing functional MRI scans at pre- and post-intervention stages [27].

It should be noted that treatment sessions with VR equipment were necessarily conducted in a supervised office-based setting in order to prevent the risk of injury while children move around the room with the VR goggles on [47]. Therefore, this type of intervention is not suitable for home-based practice.

##### Passive Dichoptic Movie/Video Viewing

Another type of PL amblyopia treatment that has been developed over recent years as an alternative to traditional patching is the passive binocular viewing of dichoptic movies–videos. It is based on similar PL principles with dichoptic video games, but it does not involve active participation. Patients were simply asked to focus and watch movies that have been modified for dichoptic presentation [27,29,36,40]. Researchers applied either contrast rebalancing or visual blur to create dichoptic viewing conditions, and the treatment course began with fully rebalanced movies and gradually advanced to less processed movies [29,36,40]. Depending on the study design, sessions took place at the office or at home [29,36,40,41].

A handheld gaming console, the Nintendo 3DS XL (Nintendo Corp, Kyoto, Japan), was employed for this purpose in a number of studies, as it features an autostereographic display. This display includes a parallax barrier, directing half of the pixels to project toward one eye and the rest of the pixels toward the other eye, allowing for 3-D viewing without the need to wear polarized filters or shutter glasses [29,40,63]. A variety of popular animation movies were modified with appropriate software (customized MATLAB, MathWorks, Natick, MA, USA) in order to present low-contrast images to the sound (or less amblyopic) eye as a home-based treatment for amblyopia [40].

CureSight, a home-based treatment system developed by NovaSight, Ltd. (Airport City, Israel), is another example of dichoptic passive video viewing [52]. This software, with the help of an eye-tracking device, is able to follow the patient’s gaze in order to create a real-time visual blur over the fovea of the sound eye while the patient is watching videos on a computer screen wearing anaglyph glasses [52].

Another research team, using an open-source media player (VLC media player, Weathermax) that offers a bi-color anaglyphic video playback feature, converted common movies to dichoptic and conducted an office-based treatment program [36]. Patients watched these movies on an ordinary computer monitor, wearing their corrective spectacles with the addition of red/cyan filter glasses and a fogging lens (+3.00 sphere) in front of the sound eye to create visual blur [36].

In a different setting, researchers have developed an interesting and simple method to implement dichoptic movie viewing that does not require any special equipment, deploying the characteristics of polarizing filters [41]. They provided their patients with polarized film sheets, which are cheap and readily available, and instructed them to attach a sheet on a viewing target (for example, a television or tablet screen or even illustrated books) in a certain direction. Then, a second piece of filter film was attached to their spectacles, over the sound eye, in a perpendicular axis relative to the first filter. This creates full absorption of light only at the area where the two filters are overlapping [38]. Thus, the amblyopic eye receives a clear view of the presented media, while the fellow eye receives a dark spot over the media, and the rest of the surroundings remain viewable. Hence, peripheral vision is not occluded, providing stimuli for binocular fusion, which is not the case with conventional eye patching [41]. Furthermore, rotation of the spectacle filter permits a variable amount of light to pass through, offering contrast adjustment for the fellow eye as the patient shows progress [41].

Approximately 220 children between 3 and 9 years old participated in three independent RCTs, which compared dichoptic movies to patching [40,41,52]. These studies reported that rebalanced movie viewing improved VA and stereoacuity in the study group, with stable results during follow-up [40,41,52]. Compared to patching, movie viewing seems to be equally effective [34], or even superior, in terms of VA improvement [40,41]. Patching, on the other hand, has no effect on stereoacuity. These results are consistent with other small sample studies conducted with both children and adult patients [29,36]. An interesting observation is that stereoacuity improvement in adults reached a plateau after 23 h of treatment [36]. Additionally, all studies concur that patients participating in the movie protocol showed higher compliance rates [29,40,41,52].

##### Vision Therapy

Vision therapy is a combination of orthoptic exercises and perceptual visual tasks [30,53,61]. It is a variant of PL that some authors describe as a separate category. The orthoptic component of vision therapy includes various manual instruments (like antisuppression charts, aperture rule, red-green bar reader, cheiroscopes) developed over the years to train and improve visual functions, such as vergence, accommodation, and binocular summation [30]. The PL component of vision therapy includes both manual instruments (manual rotator, Marsden ball, Wayne saccadic fixator), as well as various digital training tools, like the online training platforms described in the video games section, and it is targeted towards rehabilitation of VA, interocular balance and stereopsis [30].

Several studies investigated the efficacy of vision therapy [30,32,53,54,61]. A large retrospective study in India analyzed the data of 160 children between 4 and 13 years old who underwent vision therapy with the Bynocs training platform (Kanohi Eye Pvt., Mumbai, India) [53]. Similar to the dichoptic video games, training in this platform was performed with the use of red-blue filter glasses. Results showed an immense improvement of nearly 4 logMar lines in VA, plus improvement in binocular functions [53]. Additionally, a prospective study investigated the Bynocs platform in two age groups, children between 6 and 16 and adults, with similar results in VA and binocular functions [55]. The improvement was comparable in both age groups [55].

Another retrospective comparative study included 30 children with bilateral amblyopia over the critical period who did not respond to traditional patching treatment [30]. Researchers used mainly manual instruments in conjunction with the Eyeport vision training system. They reported excellent and long-term sustained results through a 12-month follow-up period, with a significant improvement (3 logMar lines) in VA and stereoacuity (greater than 50% from baseline), compared to the control group, which showed no progress [30].

Other studies investigated the effectiveness of vision therapy combined (consequently) with occlusion in children [32,61]. They both concluded that the combination of these treatments offers a significant visual gain in both VA and stereopsis, with no regression over long follow-up periods (12 and 24 months, respectively) [32,61]. Furthermore, when comparing outcomes to occlusion solely, vision therapy provided greater benefit, and the treatment effects were achieved within a shorter time period [30,32,61]. Researchers also noted that strabismic amblyopia is more resistant to treatment than anisometropic and that foveal fixation in strabismus is important for visual recovery, corroborating findings from previous studies [32].

Vision therapy is also beneficial for adult amblyopic patients as well [54,56]. In a prospective study, VisuoPrime training software (Neurapy Pvt., Tamilnadu, India) was used as a primary intervention to treat 30 naive patients with anisometropic amblyopia. According to the authors, this software is designed to alleviate several visual deficits, such as poor fixation, saccadic latency, and crowding effect, and to enhance eye-hand coordination, pursuit accuracy, and accommodation performance [54]. Applying dichoptic principles, targets of the training tasks were presented to the amblyopic eye, while the application’s background was presented to the fellow eye [54]. Results yielded notable improvement in VA and stereopsis [54].

##### Transcranial Electric Stimulation (tES)

The concept of applying electrical current through electrodes attached to the patient’s scalp in order to excite neural responses is more than a century old. Only recently, though, electric stimulation has been combined with visual PL [66]. Both techniques have been used separately to instigate neuroplasticity in cortical areas involved in visual perception. As recent data indicate, a combination of the two produces cumulative effects [66].

Transcranial electric stimulation includes different protocols with the use of low-amperage electric current, either direct (DC) or alternative current (AC). In DC, the current flows in a single direction, and the excitatory or inhibitory effect depends on the positioning of each polarity electrode. In AC, the flow direction is constantly alternating. The amount of current used in tES is insufficient to trigger neural activation by itself, but it alters the resting potential of the neurons’ cellular membrane, thus rendering them more responsive to incoming stimuli [66]. Electric stimulation has been successfully used to intensify the training effects of PL in visual functions, such as CS, motion, and directional discrimination, as well as to alleviate the impact of crowding [66].

### 3.2. Predictive Factors for Perceptual Learning Treatment Outcome

As mentioned, there are certain amblyopic patients that are resistant to conventional occlusion therapy. Likewise, not all patients respond equally to PL as well. Several studies have investigated possible factors affecting treatment effectiveness and their impact on visual outcomes using correlation analysis models, including ANCOVA (Analysis of Co-Variance) [20,32,51,65]. Table 2 presents the factors that have been studied and their association with the final outcomes of visual PL therapy.

### 3.3. Advantages and Disadvantages of Perceptual Learning Treatment

Robert Hess summarized the main advantages and disadvantages of PL treatment in the form of dichoptic games [13].

#### 3.3.1. Advantages

- Addresses multiple amblyopia impairments: A great number of studies support that the various PL treatments are effective in improving VA of the amblyopic eye, with superior, or at least equal results compared to patching [27,37,40,54,57,58,61,67]. Furthermore, the PL approaches have a definite advantage in restoring other visual deficits, like stereoacuity, CS, and crowding effect, as well as visuomotor deficits, while patching has no impact on them [13,27,28,31,34,37,40,44,46,49,54,57,59,61].
- More entertaining: Playing video games is definitely more appealing and pleasant for young patients than wearing an eye patch [13]. By incorporating game principles (scenario- storyline, targets and enemies, levels of increasing difficulty with goals and rewards), therapy becomes more intriguing [28]. Similarly, watching animated movies in 3D mode is more exciting than watching 2-dimensionally with one eye also covered.
- No social stigma: Many patients wearing a visible-to-others eye patch or foggy spectacle report low self-esteem, depression, frustration, feelings of isolation, and poor social acceptance [13]. This burden is often the reason for poor compliance in the occlusion treatment [13]. On the other hand, children attending PL activities do not experience similar psychological stress.
- Activity log: PL software applications include a built-in log that records playing hours plus patient’s performance data, stored either in the app or on a server/cloud, therefore providing an objective track of compliance to the training program [28,29,34,45,54,65].
- Faster treatment effects: Many studies have pointed out that PL programs generate equivalent visual outcomes in a shorter time (at least 5-fold faster) compared to patching [13,27,30,34,47,50,58,61]. Thus, this improves adherence and brings optimal results [49,52]. Additionally, when PL is combined with electric brain stimulation, the improvement rate is even greater, reducing the number of sessions required to achieve therapeutic results [66].
- Sustainable effects: Studies report stable results for PL treatments, lasting long after treatment cessation [13,23,51], even 6 months later [45]. A recent prospective cohort study assessed the risk of recurrence after contrast-rebalanced dichoptic treatment in 100 effectively treated children and reported a 28% regression risk up to 3 years follow-up [68]. The secondary analysis found a similar recurrence risk to the successfully treated children with patching or atropine at 12 months (24%) [68]. Further long-term follow-up studies are needed, though, to verify these preliminary results.
- Beneficial for children, adults, and cases resistant to occlusion: PL has shown favorable results in children within and above the critical period, as well as in adults, where patching is not effective [33,51,61]. Furthermore, PL seems to offer some benefit to patients who were unresponsive to occlusion [30,37].
- Low adverse effects rates: Reported potential adverse effects of dichoptic therapy are diplopia (double vision)-usually transient, eye strain, reverse amblyopia (worsening of VA in the previously better-seeing eye), manifestation or worsening of pre-existing strabismus, drowsiness, and headache [29,36,52]. Persistent diplopia is quite rare, while transient diplopia is reported in approximately 15% of treated cases [29]. Strabismus patients are prone to develop diplopia as a result of interocular suppression rebalance [29]. Especially those beginning treatment at an age above the critical period or adults with a history of strabismus surgery in childhood are at higher risk for intractable diplopia [29]. Other studies recorded no side effects to their participants [36,52].

#### 3.3.2. Disadvantages

- PL is more complex and costly: While occlusion treatment requires a simple and cheap eye patch, PL therapies are mainly delivered through sophisticated and expensive electronic equipment, like computers, tablets, game consoles, or even VR goggles, and they often require an internet connection [13,27,39,47]. Furthermore, there is a considerable cost for the development and modification of games or training software [47]. We have already discussed a certain approach that enables plain household screens for dichoptic PL presentation with the use of polarizing films [41].
- Additionally, while most PL programs are home-based, there are several others that are designed to be conducted in an office setting [27,47]. This involves additional costs for transportation from and to the clinic, absence of work hours for parents, as well as payment for the personnel supervising the training sessions [47].
- PL interactive video games are inappropriate for younger children: Patients under 5.5 years old lack the cognitive maturity to comprehend game tasks and settings, and lengthy sessions could be too tedious for them [13,47]. Therefore, this type of treatment is not suitable for preschool children that require immediate intervention. On the contrary, passive movie viewing could be effectively applied to these patients, as they do not require understanding and interaction.

### 3.4. Compliance

As mentioned above, digital devices used for PL have the ability to record patients’ activity objectively [28,29,34,45,54,65]. Patching, on the other hand, requires parents to manually record treatment hours, which is subjective and usually over-estimated. Thus, a direct comparison between the two could be rather misleading.

Nevertheless, several studies report substantially higher adherence rates for PL compared to patching [29,33,39,45,49,52]. These rates often exceed 80% [29,39,45]. This could be attributed to the gamification of treatment, which makes it more enjoyable and motivating [45].

Other studies, though, report more conservative compliance rates, not significantly higher than those of patching [13,37,47]. Poor adherence in these cases could be due to a loss of interest in the game, either because the game design itself was not attractive enough or because children easily get bored repeating the same task [13,37,47]. According to certain authors, boredom was apparent in all age categories [47]. Age proves to be a limiting factor for compliance, as preschool children are not capable of understanding and fulfilling game tasks, and furthermore, they cannot retain their attention for too long, as already mentioned [13,47].

## 4. Discussion

As Robert Hess argued, the human visual system has a binocular structure; therefore, it is fundamentally wrong to treat it in a monocular fashion, while scientific evidence suggests that the primary deficit in amblyopia is the loss of binocularity due to interocular suppression and that monocular deficits are probably the consequence [13]. Additionally, there are important real-life benefits from restoring binocular vision rather than striving to improve VA in each separate eye without achieving fusion. Those benefits account for in-depth perception, better reading performance, adequate visuomotor coordination with application in sports activities and driving, as well as more efficient peripheral detection of approaching hazards [12,13,62].

While amblyopia is an ocular pathology arising during childhood, it is of significant importance to apply a treatment that, beyond being effective, has to be pleasant and intriguing for young patients as well. Thus, gamification of the treatment is an excellent method to help children have fun while treating their lazy eyes at the same time [37,40]. Many PL programs are based on that concept, but the final designs are probably not quite successful so far. Poor compliance, as reported in a number of studies, still remains an issue. As previously discussed, loss of interest is an important reason for that. Therefore, PL training programs and video games should be designed appropriately to provide a variety of engaging training tasks, taking into account the diverging interests of children in different age groups [47]. Younger children need more simplistic content with minimal stimuli in order to stay focused, while older children require a more complex gaming environment with greater variety and action in order to maintain their interest [31]. Dichoptic movie viewing protocols offer a good alternative to video games, providing all sorts of age-appropriate content [40].

It is accepted that PL therapies exhibit higher dose-response ratios compared to occlusion [37]. From that standpoint, studies comparing PL and occlusion programs of the same duration might provide misleading conclusions. Further studies should be conducted to increase the power of the existing scientific data supporting the efficiency of perceptual learning as a treatment for amblyopia, to determine the minimum dosage required to achieve therapeutic outcomes, and to verify the long-term durability of its effect.

## 5. Conclusions

Assessment of recent studies has shown that perceptual learning is an effective treatment for amblyopia. Network meta-analyses confirm its efficacy [7,67]. PL delivers similar or superior results compared to patching in visual acuity gain [7,60,67]. A comparison of the various PL programs indicated no differences in terms of VA improvement [7]. Thus, clinicians have a vast array of therapies as alternatives to patching at their disposal. Furthermore, PL activates neural plasticity changes, alleviates interocular suppression, and restores binocular fusion with additional benefits for stereopsis, contrast sensitivity, and other visual impairments [13,16,26,35,44,59]. Patching, on the other hand, does not improve those deficits, which are still present even in successfully treated amblyopes [62]. PL produces the same therapeutic results in less time, allowing for better compliance with the treatment plan [37]. It is also beneficial for cases unresponsive to patching and for patients beyond the critical period, as well as adults [37].

## Figures and Tables

**Table 1:** Clinical studies on perceptual learning as a treatment of amblyopia that are included in the present review.

Article	Publication Year	Geographical Region	Treatment Type	Age Ranges	Type of Amblyopia
Leal Vega et al. [27]	2022	Valladolid and Alicante, Spain	Dichoptic virtual reality (VR) video game	5–17 yo	Anisometropic
Zheng et al. [28]	2022	Shangai, China	Online PL platform versus patching	4–12 yo	Anisometropic and strabismic
Dahlmann-Noor et al. [29]	2022	London, UK	Dichoptic movie viewing versus patching	3–8 yo	Anisometropic, strabismic, and combined
Hsieh et al. [30]	2022	Taiwan	Combined vision therapy and patching versus patching alone	7–10 yo	Anisometropic
Zhong et al. [5]	2022	China	Combined PL and patching and patching alone	>7 yo	Deprivation amblyopia
Godinez et al. [12]	2021	California, USA	Dichoptic VR video game	18–62 yo	Mainly strabismic
Hou et al. [25]	2022	San Fransico, USA	Dichoptic PL video game	22–62 yo	Anisometropic, strabismic, and combined
Hou C. [31]	2022	USA	Perceptual learning with dichoptic training	22–62 yo	Anisometropic, strabismic, and combined
Milla et al. [32]	2022	Alicante, Spain	Combined vision therapy and occlusion	7–18 yo	Anisometropic and strabismic
Handa et al. [33]	2022	Japan, India	Dichoptic video game versus patching	3–12 yo	Anisometropic
Ojiabo et al. [34]	2022	South Africa	Dichoptic video game	19–29 yo	Anisometropic
Du et al. [26]	2023	Beijing, China	Augmented reality (AR)	>18 yo	
Huang et al. [30]	2022	China	PL Vision therapy	7–10 yo	Bilateral amblyopia
Tan et al. [35]	2022	China	VR versus AR video game	4–10 yo	Refractive unilateral and bilateral amblyopia
Mirmohammadsadeghi et al. [36]	2022	Tehran, India	Dichoptic movie viewing	17–37 yo	Anisometropic, strabismic, and combined
Hernández-Rodríguez et al. [37]	2021	Alicante, Spain	Monocular PL therapy versus patching	5–11 yo	Anisometropic and strabismic
He et al. [38]	2023	China	Monocular PL	10–24 yo	Anisometropic and strabismic
Pang et al. [39]	2020	Hong Kong	Dichoptic video game	>7 yo	Anisometrpic, strabismic, and combined
Jost et al. [40]	2022	Texas, USA	Dichoptic movie viewing versus patching	3–7 yo	Anisometropic, strabismic, and combined
Iwata et al. [41]	2022	Japan	Dichoptic movie viewing versus patching	4–6 yo	Anisometropic
Manny et al. [42]	2022	USA	Dichoptic video game versus spectacle correction	4–6 yo	Anisometropic, strabismic, and combined
Lee et al. [43]	2020	USA	Dichoptic video game versus monocular PL versus combination with patching, Crossover	8–18 yo	Anisometropic and strabismic
Roy et al. [44]	2022	India	Dichoptic video game versus occlusion	5–15 yo	Anisometropic
Martín-González et al. [45]	2020	Spain	Dichoptic video game	7–14 yo	Anisometropic and strabismic
Liu et al. [46]	2021	China	Dichoptic video game	6–17 yo	Anisometropic, strabismic, and combined
Kadhum et al. [47]	2021	The Netherlands	Dichoptic VR video game	4–12 yo	Anisometropic, strabismic, and combined
Molina-Martín et al. [48]	2023	Spain	Dichoptic VR video game	8–14 yo	Anisometropic
Xiao et al. [49]	2021	USA	Dichoptic movie viewing	4–12 yo	Anisometropic, strabismic, and combined
Kadhum et al. [50]	2023	The Netherlands	Dichoptic video game versus occlusion	4–12 yo	Anisometropic, strabismic, and combined
Banko et al. [51]	2023	Hungary	Dichoptic video game	6–43 yo	Anisometropic, strabismic, and combined
Wygnanski-Jaffe et al. [52]	2023	Israel	Dichoptic movie viewing	4–9 yo	Anisometropic, strabismic, and combined
Abdal et al. [53]	2022	India	Dichoptic online platform	4–13 yo	Anisometropic and isometropic
Murali et al. [54]	2021	India	Dichoptic video game	18–40 yo	anisometropic
Picotti et al. [55]	2023	Argentina	Dichoptic online platform	6–60 yo	anisometropic
Bhombal et al. [56]	2020	India	Combined dichoptic therapy, vision therapy and part time patching	20–35 yo	Bilateral refractive and anisometropic
Shah et al. [57]	2022	India	Dichoptic online platform	7–21 yo	Anisometropic and strabismic
Poltavski et al. [58]	2023	USA	Dichoptic video game versus patching	4–18 yo	Anisometropic
Lan et al. [59]	2023	China	Vision therapy	5–8 yo	Refractive
Zhu et al. [60]	2023	China	Dichoptic movie viewing versus full-time and part-time patching	4–9 yo	Anisometropic

**Table 2:** Predictive factors for final visual rehabilitation after PL therapy.

Factor	Correlation with Post-Treatment Visual Outcomes
Type of amblyopia	Strabismic amblyopia patients exhibit slower recovery and inferior final binocular functions compared to anisometropic amblyopia [32].
Age	No effect in restoration of stereopsis [51,65].
Astigmatism	The presence of astigmatism is a significant limiting factor for both near and distant VA recovery in children. Conversely, astigmatism has no such influence in adult patients [32].
Baseline stereoacuity	Strongly correlated with the improvement of stereoacuity [51,65]. Patients with poor initial stereopsis seem to require longer treatment to achieve certain degree of improvement [65]. Children with no measurable stereopsis have a >2-fold increased risk for persistent amblyopia [16].
Baseline fixation stability	Poor fixation stability is related to poor monocular and binocular functions in individuals with amblyopia. Recovery of stereopsis is only possible with stable fixation, regardless of age, etiology, or depth of amblyopia [51]. The presence of fixation eye movements (FEMs) and their amplitude, fusion maldevelopment nystagmus syndrome (FMNS), or nystagmus without FMNS in amblyopic patients are associated with weaker response to treatment and limited improvement in stereoacuity [20,51].
Baseline contrast sensitivity (CS)	Initial CS scores have the strongest effect on the final CS gain [51]. Non-measurable baseline stereoacuity and poor final distant VA were found to be restricting factors for CS improvement [51].

## Data Availability

No new data were created or analyzed in this study. Data sharing is not applicable to this article.
